# Guiding light with surface exciton–polaritons in atomically thin superlattices

**DOI:** 10.1515/nanoph-2024-0075

**Published:** 2024-05-20

**Authors:** Sara A. Elrafei, T. V. Raziman, Sandra de Vega, F. Javier García de Abajo, Alberto G. Curto

**Affiliations:** Department of Applied Physics and Eindhoven Hendrik Casimir Institute, 3169 Eindhoven University of Technology, 5600 MB Eindhoven, The Netherlands; 172281 ICFO-Institut de Ciencies Fotoniques, The Barcelona Institute of Science and Technology, 08860 Castelldefels, Barcelona, Spain; ICREA-Institució Catalana de Recerca i Estudis Avançats, 08010 Barcelona, Spain; Photonics Research Group, Ghent University-imec, Ghent, Belgium; Center for Nano- and Biophotonics, Ghent University, Ghent, Belgium

**Keywords:** exciton–polaritons, 2D semiconductors, WS_2_, van der Waals heterostructures

## Abstract

Two-dimensional materials give access to the ultimate physical limits of photonics with appealing properties for ultracompact optical components such as waveguides and modulators. Specifically, in monolayer semiconductors, a strong excitonic resonance leads to a sharp oscillation in permittivity from positive to even negative values. This extreme optical response enables surface exciton–polaritons to guide visible light bound to an atomically thin layer. However, such ultrathin waveguides support a transverse electric (TE) mode with low confinement and a transverse magnetic (TM) mode with short propagation. Here, we propose that realistic semiconductor–insulator–semiconductor superlattices comprising monolayer WS_2_ and hexagonal boron nitride (hBN) can improve the properties of both TE and TM modes. Compared to a single monolayer, a heterostructure with a 1-nm hBN spacer separating two monolayers enhances the confinement of the TE mode from 1.2 to around 0.5 μm, while the out-of-plane extension of the TM mode increases from 25 to 50 nm. We propose two simple additivity rules for mode confinement valid in the ultrathin film approximation for heterostructures with increasing spacer thickness. Stacking additional WS_2_ monolayers into superlattices further enhances the waveguiding properties. Our results underscore the potential of monolayer-based superlattices as a platform for visible-range nanophotonics with promising optical, electrical, and magnetic tunability.

## 1 Introduction

Surface polaritons are electromagnetic surface waves that can mediate enhanced light–matter interaction at the nanoscale. They are crucial for developing highly miniaturized and efficient optical devices such as modulators, sensors, light sources, and photodetectors. These waves propagate along the interface between two materials and decay in the perpendicular (out-of-plane) direction. Surface polaritons can be sustained by different types of quasiparticles in matter, like plasmons in metals, phonons in insulators, and excitons in semicoductors [1]. Noble metals are common materials for supporting surface plasmon–polaritons and guiding light below the diffraction limit due to their strong light confinement capability [2], [3]. However, active tunability remains elusive because it is difficult to substantially alter the high density of free electrons in a metal.

Compared to plasmon–polaritons, surface exciton–polaritons (SEPs) are excitons that concomitantly oscillate with photons, producing a propagating surface wave bound to the interface. Exciton–polaritons have been experimentally observed in different organic [4], [5] and inorganic crystals [6], [7] with large absorption coefficients. For example, molecular J-aggregates of organic dyes [8], [9] can sustain SEPs at room temperature and create opportunities to realize novel sensors [10]. However, SEPs in those materials still have tunability limitations. As an alternative, atomically thin materials possess extreme optical properties that can be modulated while potentially giving access to the spin and valley degrees of freedom in their electronic band structures [11], [12]. Graphene and hexagonal boron nitride can indeed support plasmon– and phonon–polaritons, including modes with appealing properties such as hyperbolic polaritons that emerge due to optical anisotropy [1], [13], [14], [15], [16]. These polaritons occur, however, at terahertz and mid-infrared frequencies [17], [18].

In the visible spectral range, semiconductor monolayers of transition metal dichalcogenides (TMDs) such as WS_2_ are good candidates for guiding light using SEPs. TMD monolayers host excitons with a high oscillator strength, producing a dramatic permittivity oscillation around the exciton energy [19]. As a result, excitons in TMDs can strongly reflect electromagnetic radiation and act as atomically thin mirrors [20], [21], [22]. Interestingly, excitons can be tuned electrically, optically, magnetically, thermally, or mechanically [22], [23], [24], [25], [26], opening a promising avenue for active nanophotonic devices. Several theoretical works have dealt with the excitation of SEPs in monolayers and their coupling to nearby emitters [26], [27], [28]. A report proposed that a monolayer could support SEPs and predicted confinement to within 2 μm of the monolayer with propagation lengths exceeding 100 μm [29]. Although the near-zero thickness of the monolayer can support waveguide modes, they are loosely confined to the TMD monolayer and require a symmetric refractive-index environment. One possibility to increase confinement is patterning the monolayer into a photonic crystal, which has been demonstrated for suspended structures [30]. For unpatterned monolayers, however, the proximity of the guided mode to the light line complicates experimental detection due to the requirement for a perfectly symmetric optical environment with low scattering [31]. Furthermore, detection relies critically on achieving narrow excitonic linewidths, which can require cryogenic temperatures [32].

**Figure 1:**
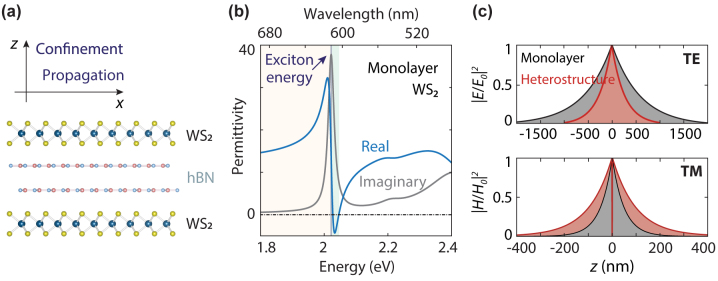
Waveguiding in WS_2_ monolayers around a permittivity oscillation due to a high exciton oscillator strength. (a) Atomically thin semiconductor–insulator–semiconductor heterostructure with a hexagonal boron nitride spacer in a symmetric refractive-index environment. (b) Experimentally retrieved permittivity of monolayer WS_2_ obtained by fitting a transmission spectrum using the transfer-matrix method and a permittivity model with 4 Lorentzians. TE or TM modes can be supported depending on the sign of the real part of the permittivity in the yellow and green areas, respectively. (c) Electric and magnetic field profiles for the modes guided by a monolayer (black) and a heterostructure with a spacer thickness of 1 nm (red) at energies of 2 and 2.0223 eV. The field is confined in the out-of-plane direction, while the waves propagate in the plane. Heterostructures contribute to increased confinement of the TE mode and reduced confinement of the TM mode.

Here, we address the fundamental challenge of guiding light bound to atomically thin semiconductors. We propose van der Waals superlattices based on semiconductor–insulator–semiconductor heterostructures to improve the propagation characteristics of surface exciton–polaritons (Figure 1(a)). We show the existence of both TE and TM guided modes and compare their dispersion relations in monolayers, heterostructures, and superlattices made of monolayer WS_2_ and hexagonal boron nitride. Compared to negligible confinement in a monolayer, we demonstrate increased confinement of the TE mode in heterostructures. Then, we clarify the impact of the thickness of the spacer layer on the guided modes. In the ultrathin film approximation, we find that the decay constants of the TE and TM modes supported by heterostructures follow simple additivity rules for their constituent layers. Additionally, we investigate the electrostatic tuning of the modes. To guide experimental realizations under different excitation conditions, we investigate the differences between two approaches for solving the mode dispersions of the superlattices using either a complex in-plane wave vector, *β*, or a complex frequency, *ω*. Our study thus produces specific directions to tailor and tune guided modes in semiconductor monolayer superlattices as a platform for nanoscale photonic and optoelectronic devices.

## 2 Strong exciton oscillator strength and permittivity

We use WS_2_ monolayers due to their strong exciton oscillator strength and narrow linewidth, which are better than in other semiconductors at room temperature and result in a record absorption coefficient. To retrieve the permittivity of a realistic, high-quality monolayer, we deposit a mechanically exfoliated WS_2_ monolayer on polydimethylsiloxane (PDMS) on a glass substrate. Using PDMS as a substrate facilitates a narrow and strong exciton peak while preserving the quantum efficiency of the monolayer emission [33]. Transmittance spectroscopy shows a strong excitonic resonance with approximately 17 % transmittance contrast and a narrow exciton linewidth, *γ*_*A*_ = 22.7 meV (Supplementary Section S1). We fit the measured transmission spectrum using transfer-matrix analysis and model the in-plane permittivity of monolayer WS_2_ with 4 Lorentzian oscillators [19], [34], [35] as $\varepsilon \left(E\right)=\varepsilon_{\text{background}}+\overset{i=4}{\underset{i=1}{\sum }}f_{i}/\left(E_{i,exciton}^{2}-E^{2}-i\gamma_{i}E\right)$ , where *E* is the photon energy, *ɛ*_background_ is the dielectric constant in the absence of excitons, and the index *i* runs over the excitonic resonances. The spectrum features peaks associated with the A and B exciton ground states, as well as the first excited state (*n* = 2) of the A exciton. The peak of the C exciton at higher energies is also included in the fitting to reproduce the overall shape of the spectrum. *E*_*i*,exciton_, *f*_*i*_ and, *γ*_*i*_ are the peak energy, oscillator strength, and linewidth of each exciton, respectively.

The permittivity oscillation around the exciton energy in Figure 1(b) is so pronounced that the real part of the permittivity, Re{*ε*}, goes from positive to negative across the excitonic resonance. Effectively, the material behaves optically like a high-refractive-index dielectric when Re{*ε*} > 0 or a reflective metal when Re{*ε*} < 0. These permittivities facilitate two regimes for guiding SEP waves: a TE mode can be supported in the range of positive and high real permittivity (above 612.5 nm, orange area), while a TM mode can be sustained where the condition Re{*ε*} + Re{*ε*_medium_} < 0 is met (from 606.5 to 612.5 nm, green area).

## 3 Surface exciton–polaritons in monolayers and heterostructures

We consider a semiconductor monolayer as a thin film of thickness *t* = 0.618 nm with permittivity *ε*_*m*_ cladded between two homogenous media with refractive indices *n*_1_ and *n*_2_. For simplicity in our analysis, we consider an isotropic thin-film model for WS_2_ to represent the material optical response. Such a layered medium can support TE and TM modes. To support a guided mode in monolayer WS_2_, however, the environment refractive index must be nearly symmetric with *n*_1_ ∼ *n*_2_. Otherwise, a cutoff appears in the minimum required TMD thickness (Supplementary Section S2). To study the mode propagation characteristics, we base our calculations on the transfer-matrix method (see Methods) [36], [37]. Through iterative transfer-matrix-method simulations using the dielectric responses of the involved materials, we model the SEP dispersion characteristics within our heterostructures. Specifically, the matrix element *M*_22_ must be zero for a guided mode. We solve the equations numerically in the complex-*ω* plane to obtain the real in-plane wave vector *β* of the supported guided mode (Supplementary Section S3) and evaluate its effective width *W*_eff_ *=* 1/Re{*q*} and effective SEP wavelength *λ*_*SE*P_ = 2π/Re{*β*}, where $q_{i}=\sqrt{\beta^{2}-\varepsilon_{i}\frac{\omega^{2}}{c^{2}}}$ is the wave vector in the out-of-plane direction in a given medium and it is known as the decay constant. This method is appropriate for guided waves in any layered system, including atomically thin superlattices.

**Figure 2:**
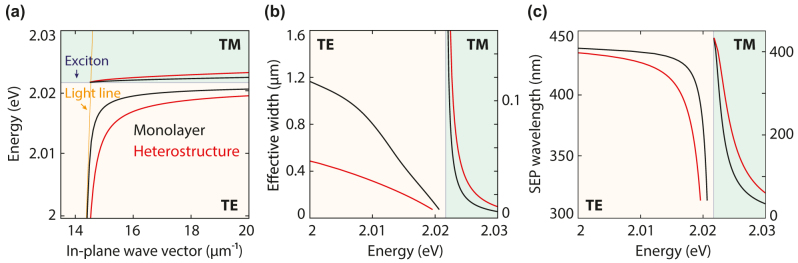
Guided modes for a WS_2_ monolayer and a WS_2_-hBN-WS_2_ heterostructure. The stack has a spacer thickness of 1 nm and is embedded in a symmetric PDMS environment. (a) Dispersion relation for the TE (yellow area) and TM (green) modes in a WS_2_ monolayer (black) and a heterostructure with 1-nm-thick hBN spacer (red) calculated using the complex-*ω* approach. Compared to the light line (orange shaded line), the TE mode is more confined in the heterostructure than in a single monolayer, while the TM mode becomes less confined. (b) Effective width of the guided modes. (c) Exciton–polariton wavelength as a function of photon energy.

We use this method first to show that a WS_2_ monolayer can support SEP modes at energies close to the exciton. SEP waves propagate along the monolayer and decay evanescently in the perpendicular direction (*z* axis in Figure 1(a)). TE and TM modes can be excited in different energy ranges depending on the sign of the permittivity. The TE mode is only supported when Re{*ε*_*m*_} > 0, while the TM mode starts to appear as the sign of the permittivity changes to negative (Figure 1(b), yellow and green areas). The TE mode of a monolayer is very close to the light line (Figure 2(a), black), with an effective refractive index close to the surrounding medium. Near the exciton energy $E_{A}^{\text{exc}}$ = 2.017 eV, the SEP wave vector becomes higher than the light line, but this mode is still only loosely confined to the monolayer. The TM mode is tightly confined to the monolayer, owing to the proximity of the propagation constant to the exciton energy line.

To overcome the confinement challenges predicted for the WS_2_ monolayer, we introduce a hexagonal boron nitride (hBN) layer between two WS_2_ monolayers. We approximate the refractive index of hBN as isotropic, setting it to 2.3 in our simulations. Although hBN is an anisotropic material [38], [39], its birefringence would only lead to minor modifications in our study. In general, the introduction of an hBN spacer layer significantly alters the dispersion relation of the TE and TM modes. In this heterostructure, the bending of the dispersion curve starts further away from the exciton energy compared to the monolayer and evolves more slowly with energy (Figure 2(a), red). This enhanced mode confinement facilitates experimental observation because otherwise loosely bound guided waves are easily scattered by imperfections.

The mode profiles for a WS_2_ monolayer (Figure 1(c), black lines) and a heterostructure (red) for the TE and TM modes at energies of 2 and 2.0223 eV, respectively, illustrate optical confinement close to the monolayer. All modes show evanescent behavior outside the waveguide core, with the TM mode being more confined than the TE mode. Using a heterostructure with an hBN spacer thickness of 1 nm also reshapes the waveguiding characteristics. When transitioning from a monolayer to a heterostructure at an energy of 2 eV, the effective width of the TE mode is compressed from 1.2 to approximately 0.5 µm (Figure 2(b)). For the TM mode at energies above the exciton peak, the mode exhibits the opposite behavior and becomes less tightly confined, with the TM-mode effective width increasing from 47 to 90 nm for a heterostructure at an energy of 2.023 eV.

## 4 Contribution of the spacer to confinement

**Figure 3:**
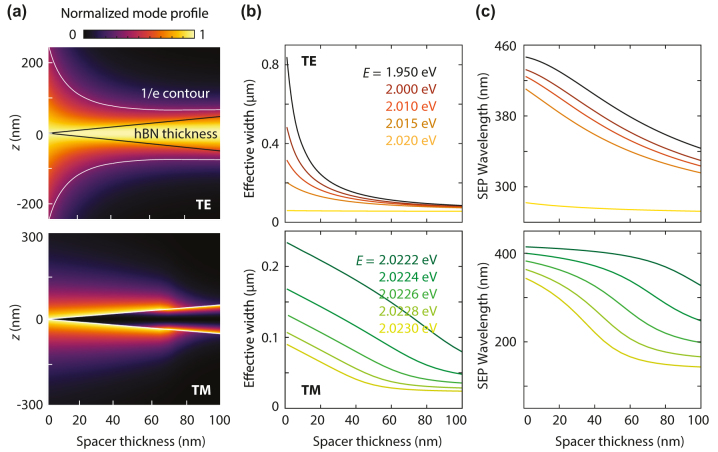
Guided-mode properties in a heterostructure as a function of spacer thickness. (a) Electric-field intensity profile for increasing spacer thickness in the TE mode at a photon energy of 2 eV and in the TM mode at 2.0223 eV. The dark-gray lines indicate the hBN thickness. The white contour line identifies confinement for 1/*e* intensity decay. (b) TE- and TM-mode effective width at different energies demonstrating the contribution of the spacer thickness to confinement. (c) Corresponding exciton–polariton wavelength, which decreases as the spacer thickens.

In heterostructures, the confinement of the guided mode depends on the insulator spacer thickness. Increasing the spacer thickness typically increases the propagation constant of the TE mode. Furthermore, the TM dispersion line moves toward the monolayer curve. To gain insight into these modes, we evaluate the intensity modal profile for heterostructures with varying spacer thickness (Figure 3(a)). The confinement of the TE mode at *E* = 2 eV is enhanced by an order of magnitude as the spacer thickness goes from 1 to 100 nm. The TE mode shifts to higher *β* as the spacer thickness increases, resulting in a more confined SEP width and a shorter SEP wavelength (Figure 3(b) and (c)). This apparent confinement is, however, due to the introduction of a material with a higher refractive index than the surrounding medium, which shifts the dispersion curve away from the PDMS light line toward that of the spacer material. Similarly, for the TM mode, increasing the spacer thickness reduces the width, resulting in higher confinement and shortening of the SEP wavelength (Figure 3(b) and (c)). Note that the TM-mode intensity profile at *E* = 2.0223 eV (Figure 3(a)) corresponds to an antisymmetric electric field distribution (Supplementary Section S4).

**Figure 4:**
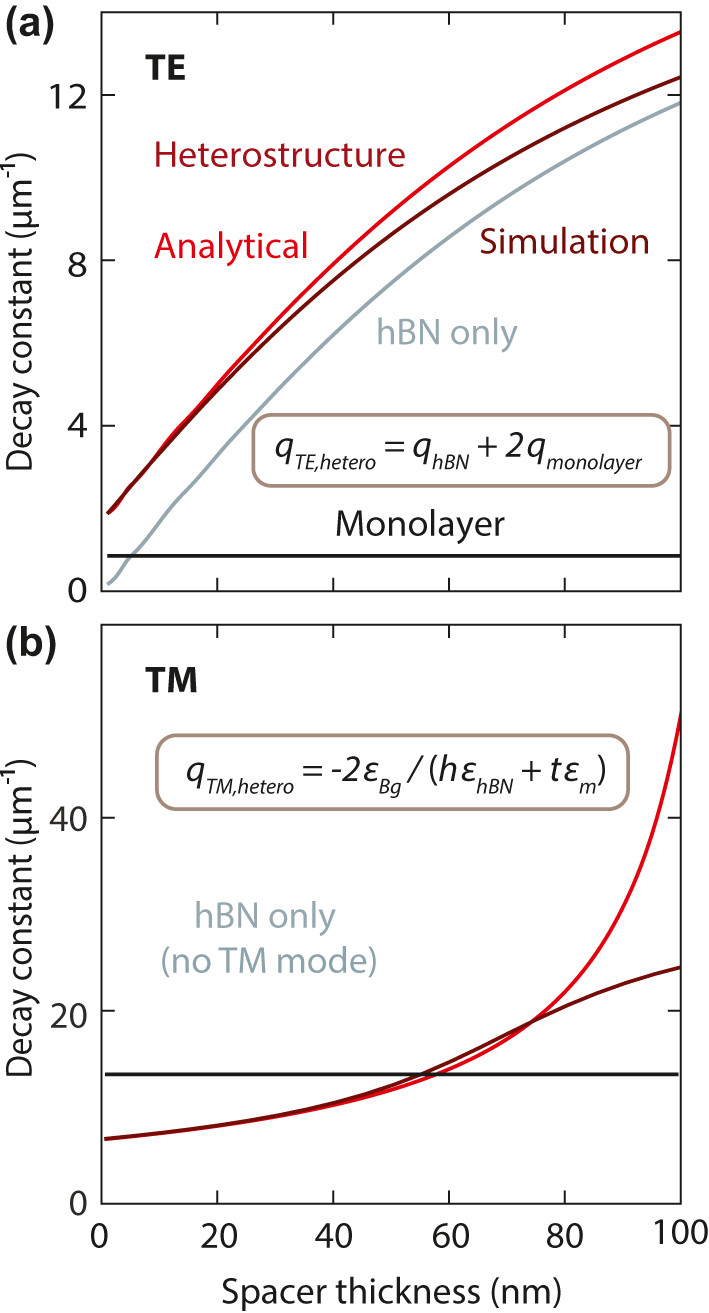
Additivity rules for the decay constants of the guided modes in a heterostructure. Comparison between the decay constant obtained from numerical simulations (dark red) and the theoretically calculated decay constant (red) using the analytical additivity rules for (a) the TE mode at *E* = 2 eV, and (b) the TM mode at *E* = 2.0223 eV. Both modes show excellent agreement for thin spacers.

Next, we compare the behavior of the guided mode in a single monolayer, a heterostructure, and the hBN spacer alone, all embedded in a symmetric dielectric environment (Figure 4). Using the ultrathin film approximation, we explicitly calculate the dependence of the decay constant of the heterostructure, *q*_hetero_, on the constituent layers for both modes. For the TE mode, the heterostructure decay constant follows a simple additivity rule of the decay constants of the individual layers, namely *q*_hBN_ and *q*_monolayer_, given by *q*_TE,hetero_ *= q*_hBN_ *+* 2 *q*_monolayer_ (proof in Supplementary Section S5). For the TM mode, instead, the heterostructure decay constant is described by *q*_TM,hetero_ = −2 *ε*_Bg_ */*(*h ε*_hBN_ *+ t ε*_monolayer_), where *ε*_Bg_ denotes the permittivity of the background medium, *h* and *t* represent the thicknesses of the hBN layer and the semiconductor monolayer, respectively, and *ε*_hBN_ and *ε*_monolayer_ are their corresponding permittivities (proof in Supplementary Section S5). These two additivity rules for TE and TM modes demonstrate the simple but distinct relations between the permittivities and thicknesses of the constituent layers and confinement in heterostructures.

We analyze first the behavior of the decay constant for the WS_2_ monolayer and hBN layers alone. The decay constant for the monolayer is a horizontal black line in Figure 4(a) and (b), as there is no spacer. If we consider an hBN film only, it supports a TE mode with increasing confinement for increasing thickness (gray line in Figure 4(a)). Conversely, the TM mode is absent for hBN alone at this photon energy due to its positive refractive index (no gray line in Figure 4(b)). For complete heterostructures containing both WS_2_ and hBN, we observe an excellent agreement between the decay constants obtained using the analytical additivity rules (red lines in Figure 4(a) and (b)) and the numerically simulated decay constants (dark red), particularly for small thicknesses below a few tens of nanometers.

## 5 Engineering the guided modes in superlattices

**Figure 5:**
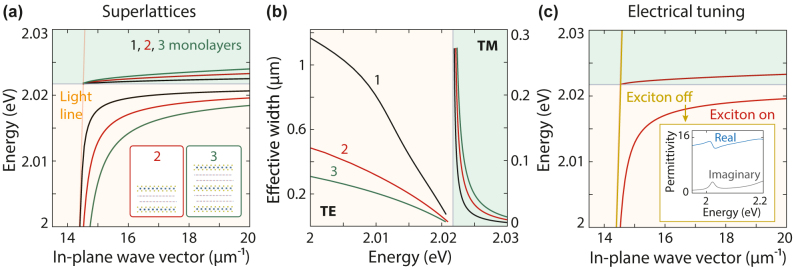
Engineering the dispersion of guided modes in superlattices. (a) Superlattices of WS_2_ monolayers in stacked heterostructures with hBN spacer thickness of 1 nm and a symmetric PDMS environment. In-plane wave vector as a function of mode energy for structures with one (black), two (red), and three semiconductor monolayers (green). (b) Comparison of the effective widths of the structures in (a), showing a modal compression (TE) or expansion (TM) as the number of layers increases. (c) Predicted tunability when the exciton peak is electrically weakened in a heterostructure comprising two WS_2_ monolayers and a 1-nm hBN spacer surrounded by PDMS. The TE mode evolves from a confined (red) to a radiation-dominated wave (gold). The TM mode cannot be supported in the absence of a strong exciton. Inset: In-plane permittivity when the exciton is off.

A superlattice geometry – a heterostructure stack – can further improve the mode confinement and make the SEP properties more appealing for nanophotonics. The TE mode moves away from the light line for superlattices, providing higher confinement for an increasing number of monolayers (Figure 5(a) and (b)). The effective TE-mode width is 1.2, 0.5, and 0.3 μm for one, two, and three monolayers, respectively. The TM-mode width at *E* = 2.023 eV rises to 100 nm with three monolayers, suggesting reduced confinement of the TM mode within the structure. The dispersion line moves away from the exciton peak energy as we go from one to three monolayers (Figure 5(a) and (b)).

To exploit the advantageous tunability of SEPs, we evaluate how the electrical control of the A exciton can allow active tuning of the guided mode. The refractive index of monolayer TMDs can be tuned using electrical gating; carrier injection can tune and broaden the in-plane permittivity around the exciton resonance [23]. To incorporate tunability in our simulations, we suppress the excitonic behavior of the WS_2_ monolayers by reducing the oscillator strength from 1.6 to 0.1 eV^2^, resulting in a drop of ∼50 % of its original permittivity near the A exciton (Figure 5(c), inset). Although electrical doping can induce a slight shift in the exciton resonance energy, accompanied by linewidth broadening [23], such a shift would not qualitatively alter the dispersion calculations. We thus simplify the tuning process by adjusting the oscillator strength alone, as it has a more pronounced impact on our calculations. With this modified permittivity, we can control and potentially modulate the guided modes (Figure 5(c)). The TE mode confinement is frustrated in the heterostructure after suppressing the exciton by turning it toward the light line. Simultaneously, electrical tuning eliminates the possibility of sustaining the TM mode altogether because the permittivity is now positive in the energy range where a strong exciton produced a negative permittivity. Therefore, both modes show promise for modulation.

## 6 Approaches to solve the dispersion relation: complex *β* and complex *ω*

The conditions under which SEPs can be observed in heterostructures depend on the experimental configuration. The governing equations of the guided modes are defined in the complex plane. Consequently, it is possible to solve the dispersion relation by finding the zeros of the transfer-matrix element *M*_22_ in the complex-wave-vector plane or the complex-frequency plane, while keeping the other parameter real (see Methods) [40], [41], [42]. The complex-*β* and complex-*ω* approaches lead to different dispersion relations and describe different experimental conditions for polariton excitation [41]. The complex-*β* approach is suitable when the excitation is a monochromatic wave localized in space [40], [43], [44], [45], whereas a complex *ω* describes better pulsed or broadband excitation at a fixed angle [46], [47], [48], [49], [50]. In all our results so far, we solved the guided modes using the complex*-ω* approach. Here, we compare the dispersion relations obtained using the complex-*β* and complex-*ω* approaches.

**Figure 6:**
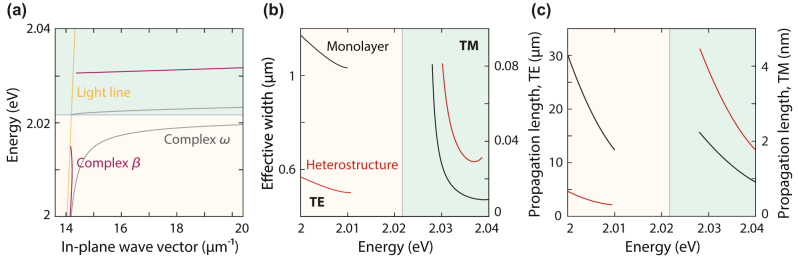
Dispersion relations in the complex-wave-vector and complex-frequency approaches. (a) Mode dispersion in a heterostructure obtained using the complex-*β* approach (purple) compared to the complex-*ω* approach (gray), corresponding to different experimental situations. Unphysical branches are not shown in the dispersion diagram. The heterostructure consists of two WS_2_ monolayers separated by a 0.3-nm-thick hBN monolayer in a symmetric PDMS environment. (b and c) Effective width and propagation length obtained by using the complex-*β* approach for both guided modes in the same heterostructure and in a monolayer.

For the complex-*ω* solutions, we observe an asymptote for large values of *β* for the TE and TM modes (Figure 6(a), gray lines). Instead, for the complex-*β* approach at a given real *ω* (purple lines), the dispersion relation of the TE mode shows a back-bending limiting the maximum value of *β*. We remove the unphysical branches from the complex-*β* results because physically meaningful solutions should have real and imaginary parts of the wave vector with the same sign. The TM-mode dispersion lines occur within different energy ranges with a shift between complex-*β* and complex-*ω* solutions. The reason for this shift is that when we keep *ω* as a real value and solve the mode equation, the obtained *β* values possess a significant imaginary part for the TM mode. However, enforcing a real *β* requires the film permittivity to be strongly negative. Negative permittivity only occurs near the exciton, which shifts the obtained real part of *ω* closer to the exciton peak. We also compare the spacer thickness dependence of the guided modes using both approaches. While the TE mode can propagate for any spacer thickness, we obtain a cutoff thickness for the TM mode for a 1-nm hBN spacer (Supplementary Section S6). Above this thickness, the effective total permittivity of the stack becomes positive, and no TM mode is supported.

Focusing on specific energies, we observe a similar evolution of the effective mode width for both approaches (Figure 6(b)). For complex *β*, the TE mode at *E* = 2 eV is confined to around 0.55 μm for the heterostructure (red) compared to 1.15 μm for the monolayer (black), whereas the TM mode at *E* = 2.03 eV expands from 30 nm for the monolayer to 80 nm for the heterostructure. Additionally, the complex-*β* approach allows us to calculate an additional SEP property: the propagation length, $L_{p}=1/\left(2\;\text{Im}\{\beta \}\right)$ . For the monolayer, the TE and TM modes can propagate for approximately 30 μm and 2 nm at energies of 2 and 2.03 eV, respectively (Figure 6(c)). For the heterostructure, the propagation length of the TE mode shortens to 5 μm at the same energy and drops rapidly as the energy gets closer to the exciton due to strong absorption related to the imaginary part of the monolayer permittivity. The TM mode propagation remains extremely dampened but improves to 4 nm.

**Figure 7:**
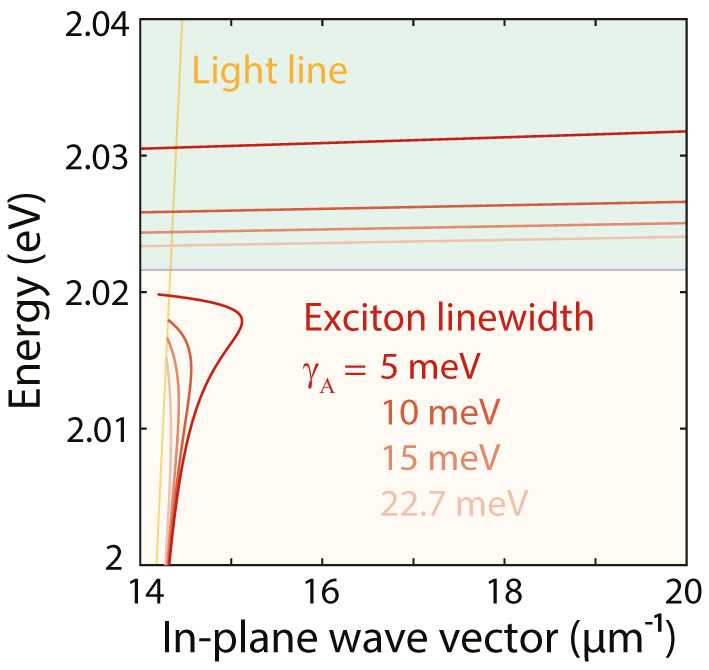
Enhanced confinement for decreasing exciton linewidth *γ*_*A*_. Exciton–polariton dispersion for a heterostructure obtained using the complex-*β* approach for linewidths *γ*_*A*_ = 5, 10, 15, and 22.7 meV, demonstrating the crucial role played by the exciton quality. The heterostructure consists of two WS_2_ monolayers separated by a 0.3-nm-thick hBN spacer (*i.e.*, one monolayer) in a symmetric PDMS environment.

Finally, we demonstrate the effect of the exciton linewidth *γ*_*A*_, which can be controlled by lowering the temperature [51], [52], on the different modes in our heterostructures. We show that decreasing the linewidth (or increasing the oscillator strength) is particularly beneficial for complex-*β* solutions of both TE and TM modes. We vary the A-exciton linewidth in our 4-Lorentzian permittivity model and calculate the SEP dispersion curve for *γ*_*A*_ = 22.7 (experimentally retrieved value at room temperature), 15, 10, and 5 meV (Figure 7). The TE mode has a more pronounced back-bending line and higher confinement using the narrowest linewidth, underscoring the need for high-quality excitons and possibly low temperatures to ease observation in experiments described by the complex-*β* approach [32]. In the complex-*ω* approach, adjustments to the linewidth do not significantly affect the dispersion. However, the propagation length exhibits changes because modifying the linewidth causes a shift in the permittivity in the complex plane, bringing it closer to the real axis.

## 7 Conclusions

We have investigated surface exciton–polaritons supported by atomically thin semiconductor–insulator–semiconductor heterostructures and their superlattices. These guided waves rely on the presence of strong exciton resonances with a high oscillator strength and narrow linewidth, as provided by WS_2_ monolayers even at room temperature. The observation of these modes also requires a symmetric optical environment. Both TE and TM modes are possible for high-quality monolayers within spectral ranges characterized by positive and negative permittivities, respectively. Compared to the monolayer modes, the heterostructure architecture modifies and controls the exciton–polariton confinement, effective width, wavelength, and propagation length. Increasing the insulator spacer thickness provides higher confinement for both TE and TM modes. Similarly, using heterostructures incorporating a larger number of monolayers and ultrathin spacers can further increase the TE mode confinement while resulting in a more delocalized TM mode. We proposed strongly controlling and modulating the guided modes by switching the monolayer excitons on and off. Finally, we have shown that surface exciton–polariton waves can be predicted with either a complex-wave-vector or a complex-frequency approach. These two approaches provide qualitatively different mode dispersions and properties. As they describe different experimental conditions, it is critical to consider the most appropriate complex-plane approach to model a specific experiment.

Our results offer substantial practical advantages in the use of surface exciton–polariton waves supported by monolayer semiconductors. TE modes have previously required a closely symmetric refractive index environment as well as patterning to achieve the necessary confinement for experimental detection. Our approach faciliates the observation of surface-exciton polaritons by enhancing confinement without the need for patterning, potentially enabling widespread implementation and broader applications. For TM modes, which have not been observed yet experimentally in semiconductor monolayers, our findings suggest that their reduced confinement could result in longer propagation lengths. However, our approach may also require greater complexity in fabrication and alignment compared to monolayer systems. In addition, the diverse tuning mechanisms of excitons in monolayer semiconductors provide a control knob for guided waves based on changes to the exciton strength, linewidth, and peak energy. For example, all-optical modulation due to lattice heating has been shown to substantially alter the reflectivity of atomically thin mirrors [22] and could be used to modulate exciton–polaritons in space and time. Based on our results and given the fast pace of developments in this area, we argue that atomically thin semiconductors hold great promise for nanoscale tunable photonics at visible wavelengths.

## 8 Methods

### 8.1 Transfer-matrix method

In our study of surface exciton–polaritons in semiconductor–insulator–semiconductor heterostructures, we employ the transfer-matrix method to theoretically analyze and simulate the propagation of electromagnetic waves at the interfaces between different media. We start by considering two different media separated by a planar interface. The forward and backward wave amplitudes in medium 1 are denoted by *A*_1_ and *B*_1_, respectively. Similarly, *A*_2_ and *B*_2_ are the waves in medium 2. The interface transfer matrix connects the amplitudes of the waves in the two media through $\left[\begin{array}{c} A_{2}\\ B_{2} \end{array}\right]=M_{2\leftarrow 1}\left[\begin{array}{c} A_{1}\\ B_{1} \end{array}\right]$ , where $M_{2\leftarrow 1}=\left[\begin{array}{cc} M_{11} & M_{12}\\ M_{21} & M_{22} \end{array}\right]$ .

By applying the electric- and magnetic-field boundary conditions depending on the polarization of the wave (TE or TM), we can evaluate the matrix elements *M*_*ij*_, which depend on the optical properties of the layered medium. For the TE mode, we obtain $M_{11}=\frac{1}{2k_{z2}}\left(k_{z2}+k_{z1}\right)$ , $M_{12}=\frac{1}{2k_{z2}}\left(k_{z2}-k_{z1}\right)$ , $M_{21}=\frac{1}{2k_{z2}}\left(k_{z2}-k_{z1}\right)$ , and $M_{22}=\frac{1}{2k_{z2}}\left(k_{z2}+k_{z1}\right)$ . For the TM mode, we have $M_{11}=\frac{1}{2\frac{k_{z2}}{n_{2}^{2}}}\left(\frac{k_{z2}}{n_{2}^{2}}+\frac{k_{z1}}{n_{1}^{2}}\right)$ , $M_{12}=\frac{1}{2\frac{k_{z2}}{n_{2}^{2}}}\left(\frac{k_{z2}}{n_{2}^{2}}-\frac{k_{z1}}{n_{1}^{2}}\right)$ , $M_{21}=\frac{1}{2\frac{k_{z2}}{n_{2}^{2}}}\left(\frac{k_{z2}}{n_{2}^{2}}-\frac{k_{z1}}{n_{1}^{2}}\right)$ , and $M_{22}=\frac{1}{2\frac{k_{z2}}{n_{2}^{2}}}\left(\frac{k_{z2}}{n_{2}^{2}}+\frac{k_{z1}}{n_{1}^{2}}\right)$ , where $k_{zi}=\pm \sqrt{\varepsilon_{i}\frac{\omega^{2}}{c^{2}}-\beta^{2}}$ is the perpendicular component and *β* is the in-plane component of the propagation wave vector relative to the interfaces between the layers.

The propagation transfer matrix in a homogeneous medium is $\left[\begin{array}{c} A_{2}\\ B_{2} \end{array}\right]=P\left[\begin{array}{c} A_{1}\\ B_{1} \end{array}\right]$ , where $P=\left[\begin{array}{cc} {\text{e}}^{\text{i}k_{z}d} & 0\\ 0 & e^{-ik_{z}d} \end{array}\right]$ accounts for the propagation phase, and *d* is the layer thickness. The complete transfer matrix is the product of the interface and propagation matrices. For example, the transfer matrix of a film waveguide based on a monolayer is *M = M*_3←2_ *P*_2_ *M*_2←1_. For a heterostructure consisting of three stacked films, the transfer matrix has the form *M* = *M*_5←4_ *P*_4_ *M*_4←3_ *P*_3_ *M*_3←2_ *P*_2_ *M*_2←1_. We require *A*_1_ *= B*_2_ *=* 0 to guarantee confinement so that the field vanishes at infinity. In addition, *k*_*zi*_ must have an imaginary component (and a vanishing real component if losses are neglected for the sake of determining the dispersion relation) to have decaying fields at the bottom and top layers. For a guided mode, the matrix element *M*_22_ should be zero. Based on this condition, we numerically solve the resulting equation to obtain the propagation constant of the mode, as well as the field distribution in each layer.

This method remains applicable for both complex-*ω* and complex-*β* approaches. The complex-*ω* approach involves finding *ω* for each real value of *β* using a permittivity defined in the complex-*ω* plane. The obtained *ω* from the mode solution is used to extend the permittivity in the complex-*ω* plane using the 4-Lorentzian model (Supplementary Sections S1 and S3). Consequently, the permittivity for the complex-ω approach encompasses two branches, one for the TE mode and another for the TM mode, each of them requiring the determination of complex *ω* values independently. Likewise, the complex-*β* approach relies on finding the real and imaginary parts of *β* with a permittivity defined at each real *ω*.

## Supplementary Material

Supplementary Material Details
