# Supplementary material for: Guiding light with surface exciton–polaritons in atomically thin superlattices
Source: Nanophotonics. 2024 May 20;13(17):3101–11. doi: 10.1515/nanoph-2024-0075 (PMC11501836; doi:10.1515/nanoph-2024-0075)
Supplement: Supplementary file 1 — Supplementary Material Details [file j_nanoph-2024-0075_suppl_001.pdf]

Supplementary Materials

Guiding light with surface exciton-polaritons in atomically thin superlattices

S. A. Elrafei[1], T. V. Raziman[1], S. de Vega[2],
F. J. García de Abajo[2,3], A. G. Curto[1,4,5]

[1] *Department of Applied Physics and Eindhoven Hendrik Casimir Institute, Eindhoven University of Technology, 5600 MB Eindhoven, The Netherlands*
[2] *ICFO-Institut de Ciencies Fotoniques, The Barcelona Institute of Science and Technology, 08860 Castelldefels (Barcelona), Spain*
[3] *ICREA-Institució Catalana de Recerca i Estudis Avançats, 08010 Barcelona, Spain*
[4] *Photonics Research Group, Ghent University-imec, Ghent, Belgium*
[5] *Center for Nano- and Biophotonics, Ghent University, Ghent, Belgium*

* Corresponding author: A.G.Curto@TUe.nl

Contents:

Supplementary Section S1. In-plane permittivity and transmission spectrum of WS_2

Supplementary Section S2. Symmetry requirement for light guiding in atomically thin waveguides

Supplementary Section S3. Finding the guided modes

Supplementary Section S4. Field distribution for TE and TM modes in heterostructures

Supplementary Section S5. Additivity rules for out-of-plane momentum in heterostructures

Supplementary Section S6. Influence of the insulator spacer on the guided modes for the complex-ω and complex-β approaches

Supplementary Section S1. In-plane permittivity and transmission spectrum of WS_2

Sample fabrication. We mechanically exfoliate WS_2 from a synthetic crystal (HQ Graphene) using tape (SPV 9205, Nitto Denko Co.) on optically transparent polydimethylsiloxane films (PDMS, Gel-Pak PF-80-X4) deposited on glass substrates.

Optical measurements. We measure the transmission spectrum at room temperature using Köhler illumination through a microscope objective (20x Nikon CFI Plan Fluor ELWD, NA = 0.45). The signal is sent through an optical fiber to a spectrometer (Andor Shamrock 330i, with an Andor Newton 970 EMCCD camera cooled to −75 °C). We retrieve the in-plane permittivity by fitting the transmission spectrum using the transfer-matrix method and a superposition of 4 Lorentzian oscillators, namely: $\varepsilon(E) = \varepsilon_{\mathrm{B}} + \sum_{i=1}^{i=4} f_i/(E_{i,\ \mathrm{exciton}}^2 - E^2 - i\gamma_i E)$, where ε_{B} is a background permittivity, f_i is the oscillator strength of the exciton with subindex i, $E_{i,\ \mathrm{exciton}}$ is the corresponding exciton peak energy, γ_i is the linewidth of the exciton absorption band, and $E = \hbar\omega$ is the photon energy.

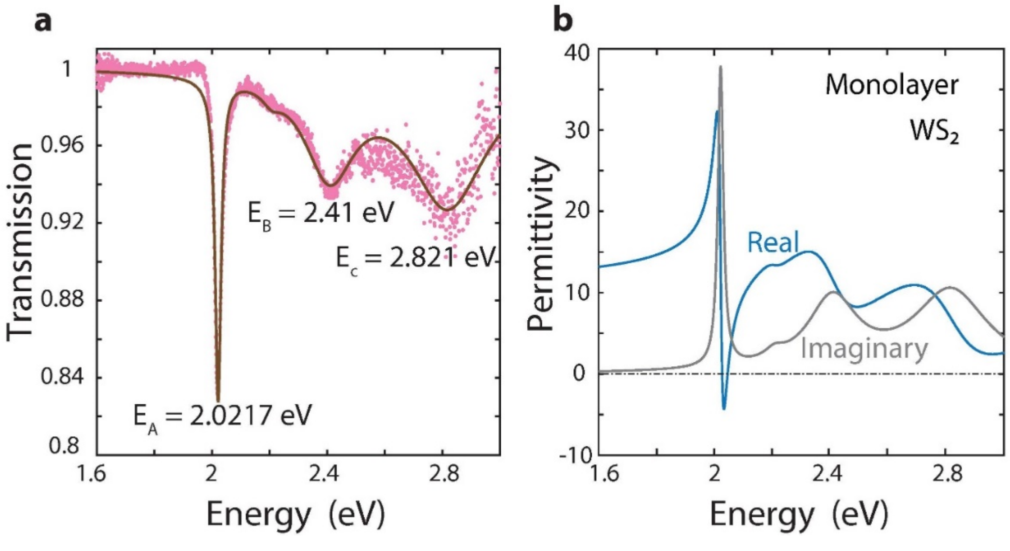

Supplementary Figure S1 | Retrieving the in-plane permittivity of monolayer WS_2. a, Experimental transmission spectrum of monolayer WS_2 on PDMS (pink) and fitted spectrum (brown). **b**, Retrieved in-plane permittivity of monolayer WS_2 obtained by fitting the transmission spectrum using the transfer-matrix method and a permittivity model with 4 Lorentzians.

Supplementary Section S2. Symmetry requirement for guiding light in atomically thin waveguides

We examine a slab waveguide model that includes a monolayer of WS_2, characterized by a refractive index n_{ML} and surrounded by media with refractive indices n_1 and n_2 (Supplementary Figure S2a). We aim to understand how differences in these refractive indices impact the cutoff thickness for both the transverse electric (TE) and transverse magnetic (TM) modes, which is a critical parameter that defines the minimum thickness at which the waveguide can support a given mode. By solving Maxwell's equations for both modes, we find the cutoff thicknesses in Supplementary Equations S1 and S2:

$$h_{\text{cutoff, TE}}^{\text{monolayer}} = \frac{\tan^{-1}\left[\left(\frac{n_2^2 - n_1^2}{n_{\mathrm{ML}}^2 - n_2^2}\right)^{\frac{1}{2}}\right]}{(n_{\mathrm{ML}}^2 k^2 - n_2^2 k^2)^{\frac{1}{2}}} \qquad \text{S1}$$

$$h_{\text{cutoff, TM}}^{\text{monolayer}} = \frac{\tan^{-1}\left[\frac{n_{\mathrm{ML}}^2}{n_1^2}\left(\frac{n_2^2 - n_1^2}{n_{\mathrm{ML}}^2 - n_2^2}\right)^{\frac{1}{2}}\right]}{(n_{\mathrm{ML}}^2 k^2 - n_2^2 k^2)^{\frac{1}{2}}} \qquad \text{S2}$$

In the case of a symmetric slab waveguide with $n_1 = n_2$, the cutoff thicknesses for the fundamental TE and TM modes are zero. We plot the relation between the cutoff thickness and the refractive index mismatch $\Delta n = n_2 - n_1$. The cutoff thickness for both TE and TM modes at the wavelengths of 615 and 608 nm decreases when Δn is reduced, as illustrated in Supplementary Figure S2b. The cutoff thickness dependence suggests a sensitivity of waveguiding on small changes in refractive index. In the case of a single-layer-thick slab, it is crucial to limit Δn below 5% for the TE mode to ensure light guiding. The TM mode is even more susceptible to refractive index mismatch: the mode ceases to exist for index variations of 0.006% in a PDMS environment. As the wavelength increases compared to the wavelengths above, the requirements for the symmetric index environment become less stringent for the

TE and TM modes, thus increasing the cutoff thickness at a given refractive-index mismatch (Supplementary Figure S2c).

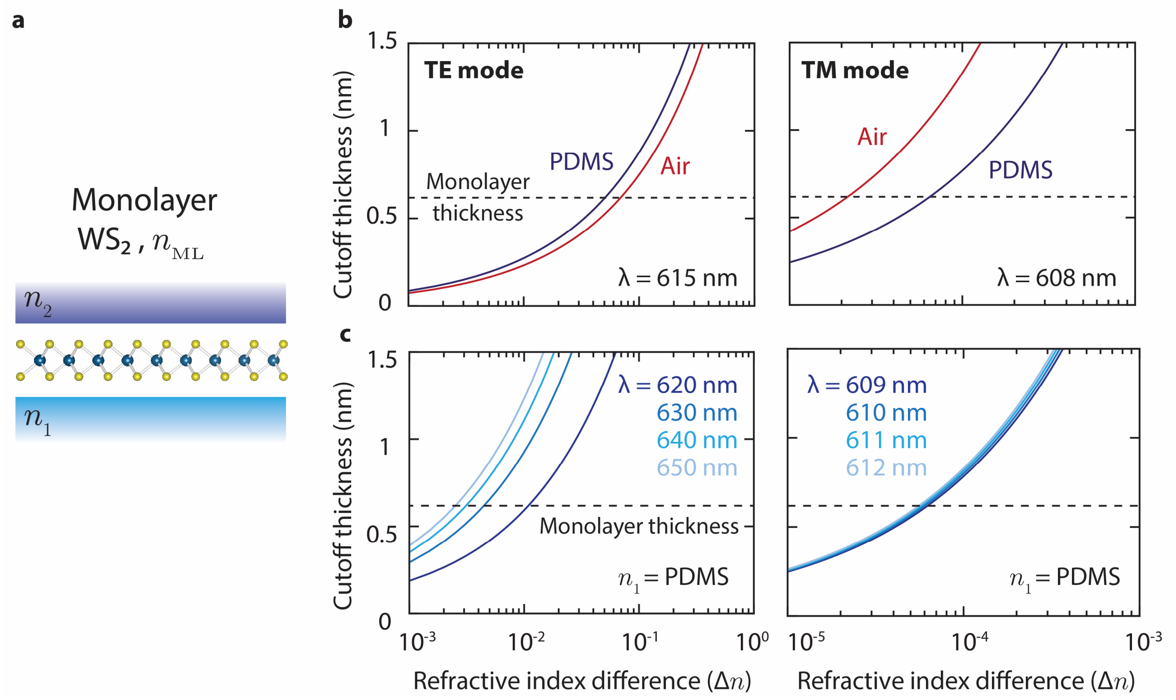

Supplementary Figure S2 | The guided mode in a monolayer is very sensitive to asymmetries in the environment refractive index. **a**, Monolayer WS_2 waveguide between two homogenous media with refractive indices n_1 and n_2. **b**, Cutoff thickness for guiding light depends on the difference in refractive indices (Δn) between the top and bottom cladding materials at a wavelength of 615 nm and 608 nm for TE and TM modes, respectively. Whe show results for n_1 corresponding to PDMS (blue) and air (red). **c**, Cutoff thickness condition at different wavelengths for both TE and TM modes when $n_1 = n_{PDMS}$.

Supplementary Section S3. Finding the guided modes

We perform numerical simulations for a layered waveguide system using the transfer-matrix method (as described in the Methods section) for monolayers and heterostructures using different complex planes. Our dispersion calculations rely on finding the correct poles in the complex plane. To find a guided mode, we search for the roots of $M_{22} = 0$ in the complex plane, which provides the first iteration of the solution for our numerical calculation. In the complex-ω approach, we sweep the complex values of ω while keeping the wave vector β real and fixed. The complex-ω plane shows the position of the zero at a particular value of β=15 μm^{-1} for the TE and TM modes (Supplementary Figure S3a, top and bottom). In contrast, in the real-valued ω approach, we tune β while ω remains constant (Supplementary Figure

S3b). Then, we use this initial solution in our simulation to evaluate the dispersion and its corresponding propagation characteristics.

This methodology remains applicable to both the complex-ω and complex-β approaches. In the complex-ω approach, the acquired ω from the mode solution is then employed to evaluate the permittivity in the complex plane, using the 4-Lorentzian model (see above). As a result, the permittivity for the complex-ω approach encompasses two branches, one related to the TE mode and another one associated with the TM mode, each requiring an independent determination of a complex ω value (Supplementary Figure S4). On the contrary, the complex-β approach involves the determination of the real and imaginary parts of β with a permittivity defined at each real value of ω (Supplementary Figure S1).

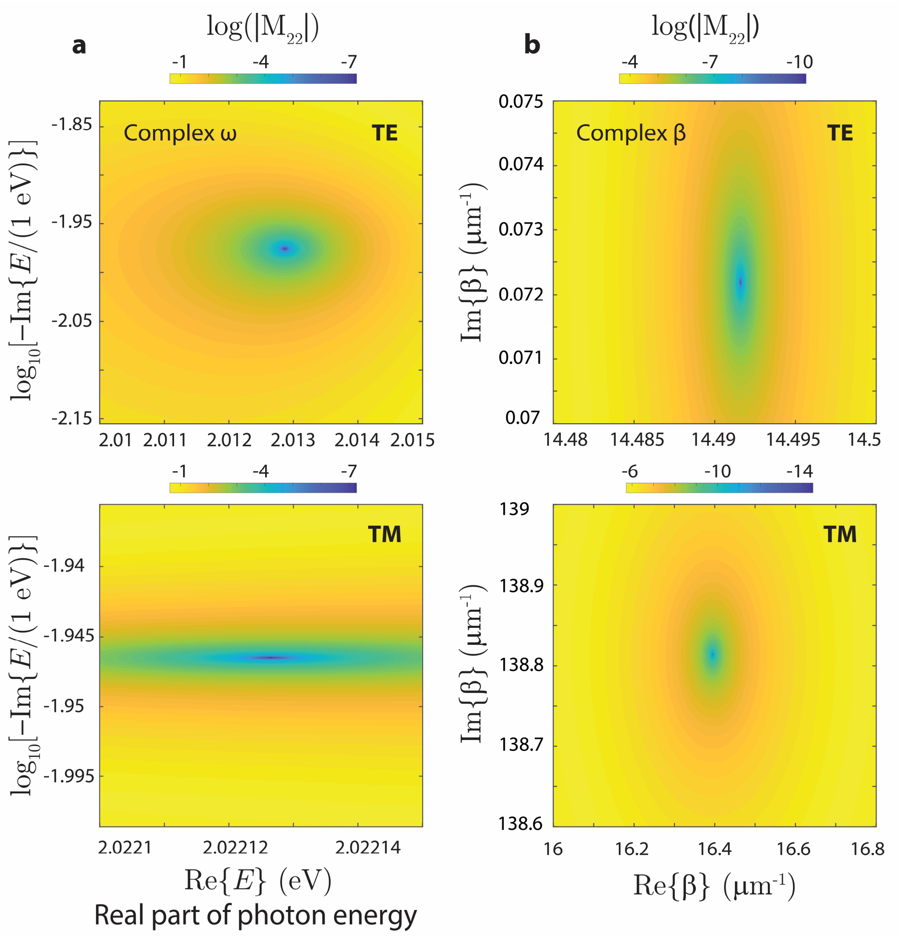

Supplementary Figure S3 | Example of the evaluation of the first-iteration solutions for TE and TM modes. Map of the M_{22} matrix element in: **a**, the complex-ω plane; **b**, the complex-β plane.

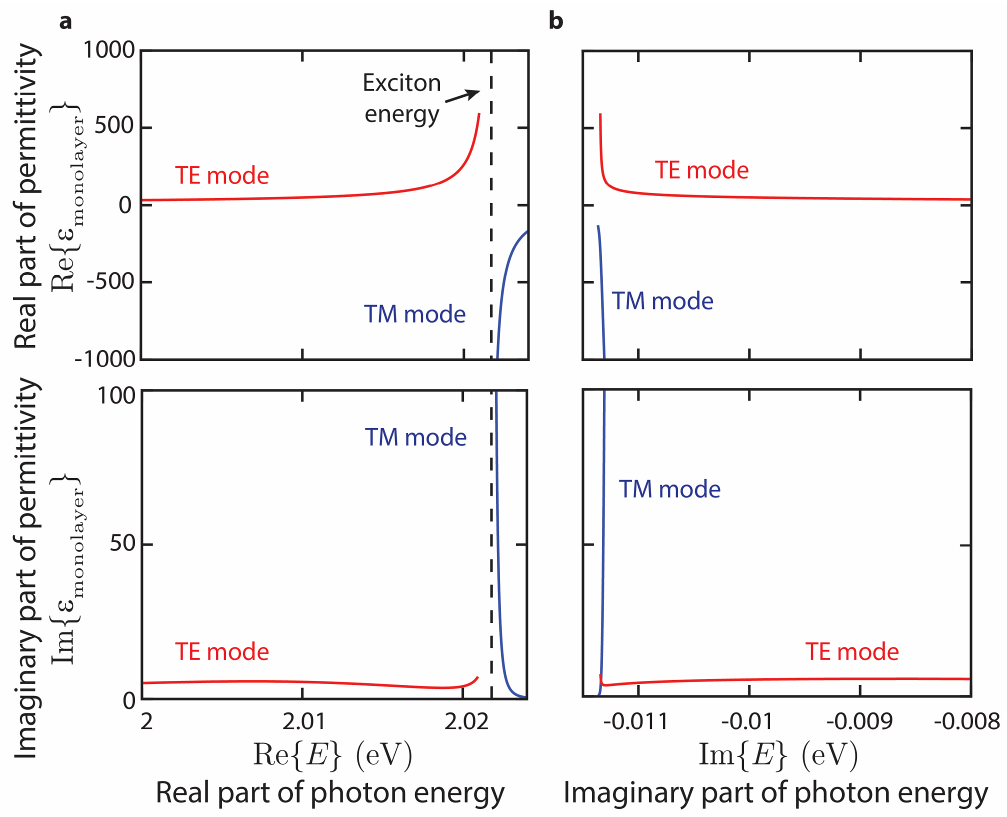

Supplementary Figure S4 | Example of permittivity of monolayer WS_2 in the complex-ω plane. a, Real and imaginary parts of the permittivity versus the real part of the photon energy $E = \hbar\omega$ in the complex-ω plane for TE and TM modes. **b**, Real and imaginary parts of the permittivity versus the imaginary part of the energy for the TE and TM solutions.

Supplementary Section S4. Field distribution for TE and TM modes in heterostructures

Using the transfer-matrix method for monolayers and heterostructures, we evaluate the field distribution of the supported TE and TM modes. These two types of waveguide modes have markedly different field distributions. For the TE mode, the normalized tangential electric field component shows a symmetric distribution at $E = 2$ eV (Supplementary Figure S5). In contrast, the TM magnetic field distribution is anti-symmetric with respect to the middle plane of the hBN spacer. This field component goes through zero at the center of the hBN film.

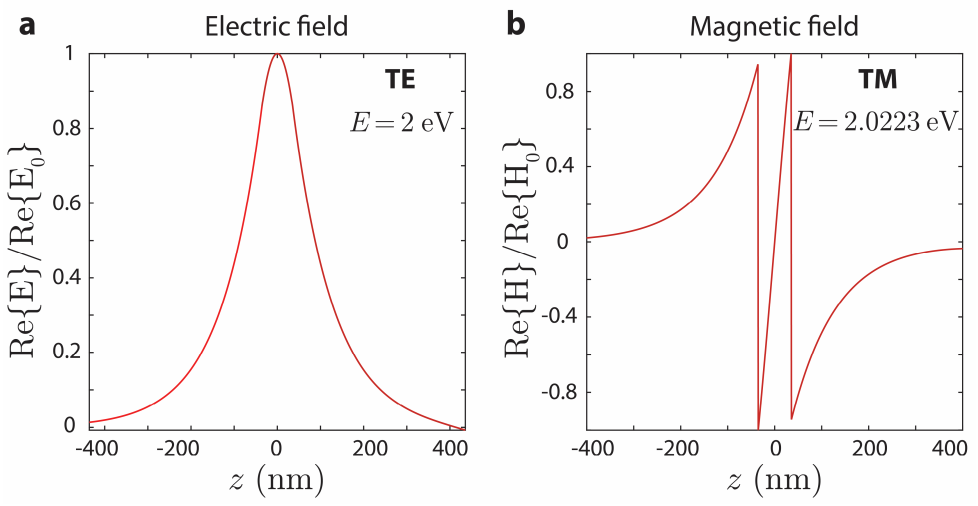

Supplementary Figure S5 | Normalized field components for a heterostructure. a, Normalized electric field for the TE mode at photon energy $E = 2$ eV. **b**, Normalized magnetic field for the TM mode at $E = 2.0223$ eV. The heterostructure has an hBN spacer thickness of 70 nm for ease of visualization.

Supplementary Section S5. Additivity rules for out-of-plane momentum in heterostructures

Consider a heterostructure composed of two monolayers with thickness $t_{\text{monolayer}}$ and surface conductivity σ, separated by a spacer material with thickness $h = 2a$ and permittivity ε_2. This heterostructure is placed in a homogenous medium with permittivity ε_1 (Supplementary Figure S6).

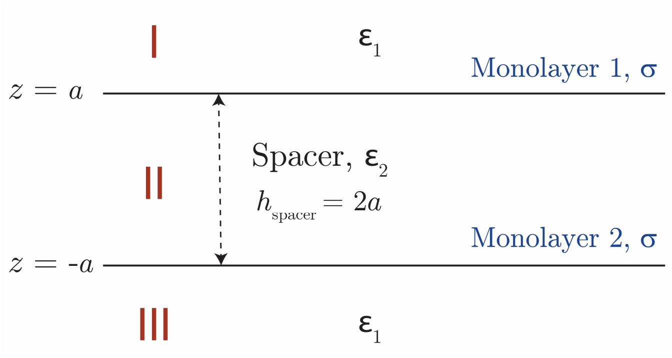

Supplementary Figure S6 | Evaluation of decay constants. Schematic of a heterostructure comprising two monolayers with surface conductivity σ, and a spacer material with thickness $h = 2a$ and permittivity ε_2. The structure is placed in a homogeneous environment with permittivity ε_1.

TE mode

For medium n in the homogeneous regions in the heterostructure and using Maxwell's equations, we can write the fields:

$$\overrightarrow{E_n} = (A_n e^{i\beta x + ik_z z} + B_n e^{i\beta x - ik_z z})\hat{y},$$

$$\overrightarrow{H_n} = \frac{-i}{\omega\mu_0}\vec{\nabla} \times \overrightarrow{E_n},$$

$$\overrightarrow{H_{n//}} = \frac{i}{\omega\mu_0}\frac{\partial E_y}{\partial z} = \frac{-k_z}{\omega\mu_0}(A_n e^{i\beta x + ik_z z} - B_n e^{i\beta x - ik_z z}),$$

$$B_I = A_{III} = 0.$$

In medium *I* and *III,* as shown in Supplementary Figure S6, we have:

$$q^2 = \beta^2 - \varepsilon_1 k_0^2 \quad \text{and} \quad k_z = iq, \qquad \text{S3}$$

where q is the out-of-plane decay constant, β is the propagation constant (or in-plane wave vector), k_0 is the free-space wavenumber, and k_z is the component of the wave vector in the out-of-plane direction (perpendicular to the propagation direction).

In medium *II*, we have: $$m^2 = \varepsilon_2 k_0^2 - \beta^2 \quad \text{and} \quad k_z = m, \qquad \text{S4}$$

where m is a parameter related to the decay constants with units of inverse length.

In the infinitesimally thin monolayer approximation, the boundary conditions at the interfaces are $E_+ = E_-$ and $H_+ - H_- = \sigma\hat{n} \times E$.

At the upper monolayer ($z = a$), we obtain:

$$A_I e^{-qa} = A_{II} e^{ima} + B_I e^{-ima} \quad \text{and}$$

$$-\frac{iq}{\omega\mu_0}A_I e^{-qa} + \frac{m}{\omega\mu_0}(A_{II}e^{ima} - B_{II}e^{-ima}) = \sigma A_I e^{-qa}.$$

At the lower monolayer ($z = -a$), we have:

$$B_{II} e^{-qa} = A_{II} e^{-ima} + B_{II} e^{ima} \quad \text{and}$$

$$-\frac{m}{\omega\mu_0}(A_{II}e^{-ima} - B_{II}e^{ima}) - \frac{iq}{\omega\mu_0}B_{III}e^{-qa} = \sigma B_{III} e^{-qa}.$$

We can then write all the boundary conditions as a matrix equation:

$$\begin{pmatrix} -1 & e^{ima} & e^{-ima} & 0 \\ -iq - \sigma\omega\mu_0 & me^{ima} & -me^{-ima} & 0 \\ 0 & e^{-ima} & e^{ima} & -1 \\ 0 & -me^{-ima} & me^{ima} & -iq - \sigma\omega\mu_0 \end{pmatrix} \begin{pmatrix} A_I e^{-qa} \\ A_{II} \\ B_{II} \\ B_{III} e^{-qa} \end{pmatrix} = 0.$$

For the mode to exist, the matrix determinant should be zero:

$$e^{-2ima}(-iq-\sigma\omega\mu_0-m)^2-e^{2ima}(-iq-\sigma\omega\mu_0+m)^2=0,$$

$$\frac{m-iq-\sigma\omega\mu_0}{m+iq+\sigma\omega\mu_0}=\pm e^{-2ima}. \qquad \text{S5}$$

Next, we investigate the decay constant for a monolayer, an hBN film, and a heterostucture:

- **Only monolayer (no hBN):** this is equivalent to setting $a=0$:

$$q=iq\omega\mu_0=2q_{\text{monolayer}}. \qquad \text{S6}$$

- **Only hBN (no monolayer):** this is equivalent to setting $\sigma=0$:

$$\frac{m-iq}{m+iq}=\pm e^{-2ima}. \qquad \text{S7}$$

The lowest-order solution, taking the positive sign, is:

$$im+q=e^{-2ima}\ (im-q),$$

$$q=im\frac{e^{-2ima}-1}{e^{-2ima}+1}=m\ \tan(ma),$$

$$q^2+m^2=(\varepsilon_2-\varepsilon_1)k_0^2=R^2.$$

Using the small-thickness approximation, Equation *S7* is satisfied when *m* ~ *R:*

$$q_{\text{hBN}}=R\ \tan(Ra) \qquad \text{S8}$$

- **Heterostructure with two WS_2 monolayers and hBN as a spacer:**

Replacing Equation S6 in Equation S5, we obtain

$$\frac{m-i(q-2q_{\text{monolayer}})}{m+i(q-2q_{\text{monolayer}})}=\pm e^{-2ima}. \qquad \text{S9}$$

Let us define $\tilde{q}=q-2q_{\text{monolayer}}$ and $\frac{m-i\tilde{q}}{m+i\tilde{q}}=\pm e^{-2ima}$. Solving in the same way as in Equations S7 and S8, we obtain

$$\tilde{q}=R\ \tan(Rh),$$

$$q=R\ \tan(Rh)+2q_{\text{monolayer}}=q_{\text{hBN}}+2q_{\text{monolayer}}.$$

TM mode

For medium n:

$$\overrightarrow{H_n}=(A_ne^{i\beta x+ik_zz}+B_ne^{i\beta x-ik_zz})\hat{y},$$

$$\overrightarrow{E_n}=\frac{i}{\omega\varepsilon_0\varepsilon}\overrightarrow{\nabla}\times\overrightarrow{H_n},$$

$$\overrightarrow{E_n}=\frac{-i}{\omega\varepsilon_0\varepsilon}\frac{\partial H_y}{\partial z}=\frac{k_z}{\omega\varepsilon_0\varepsilon}(A_ne^{i\beta x+ik_zz}-B_ne^{i\beta x-ik_zz}).$$

We use the same approach as in the TE mode:

At the upper monolayer ($z=a$):

$$\frac{iq}{\omega\varepsilon_0\varepsilon_1}A_Ie^{-qa}=\frac{m}{\omega\varepsilon_0\varepsilon_2}(A_{II}e^{ima}-B_{II}e^{-ima}),$$

$$A_I e^{-qa} - A_{II} e^{-ima} - B_{II} e^{-ima} = -\sigma \frac{iq}{\omega\varepsilon_0\varepsilon_1} A_I e^{-qa}.$$

At the bottom monolayer ($z = -a$):

$$\frac{-iq}{\omega\varepsilon_0\varepsilon_1} B_{III} e^{-qa} = \frac{m}{\omega\varepsilon_0\varepsilon_2}(A_{II} e^{-ima} - B_{II} e^{ima}),$$

$$A_{II} e^{-ima} + B_{II} e^{ima} - B_{III} e^{-qa} = \sigma \frac{iq}{\omega\varepsilon_0\varepsilon_1} B_{III} e^{-qa}.$$

Let us set $K_1 = \frac{iq}{\omega\varepsilon_0\varepsilon_1}$ and $K_2 = \frac{m}{\omega\varepsilon_0\varepsilon_2}$. We write all the boundary conditions as a matrix equation:

$$\begin{pmatrix} -1 - \frac{\sigma iq}{\omega\varepsilon_0\varepsilon_1} & e^{ima} & e^{-ima} & 0 \\ \frac{-iq}{\omega\varepsilon_0\varepsilon_1} & \frac{m}{\omega\varepsilon_0\varepsilon_2} e^{ima} & -\frac{m}{\omega\varepsilon_0\varepsilon_2} e^{-ima} & 0 \\ 0 & e^{-ima} & e^{ima} & -1 - \frac{\sigma iq}{\omega\varepsilon_0\varepsilon_1} \\ 0 & -\frac{m}{\omega\varepsilon_0\varepsilon_2} e^{-ima} & \frac{m}{\omega\varepsilon_0\varepsilon_2} e^{ima} & \frac{-iq}{\omega\varepsilon_0\varepsilon_1} \end{pmatrix} \begin{pmatrix} A_I e^{-qa} \\ A_{II} \\ B_{II} \\ B_{III} e^{-qa} \end{pmatrix} = 0.$$

For a mode to exist, the matrix determinant should be zero:

$$e^{-2ima}\left[\left(-1 - \frac{\sigma iq}{\omega\varepsilon_0\varepsilon_1}\right)\frac{m}{\omega\varepsilon_0\varepsilon_2} - \frac{iq}{\omega\varepsilon_0\varepsilon_1}\right]^2 - e^{2ima}\left[\left(-1 - \frac{\sigma iq}{\omega\varepsilon_0\varepsilon_1}\right)\frac{m}{\omega\varepsilon_0\varepsilon_2} + \frac{iq}{\omega\varepsilon_0\varepsilon_1}\right]^2 = 0,$$

$$e^{-2ima}\left[1 - \left(1 + \frac{\sigma iq}{\omega\varepsilon_0\varepsilon_1}\right)\frac{im\varepsilon_1}{q\varepsilon_2}\right]^2 - e^{2ima}\left[1 + \left(1 + \frac{\sigma iq}{\omega\varepsilon_0\varepsilon_1}\right)\frac{im\varepsilon_1}{q\varepsilon_2}\right]^2 = 0.$$

Let us also set $\frac{i\sigma q}{\omega\varepsilon_0\varepsilon_1} = \Phi$, $\frac{im\varepsilon_1}{k\varepsilon_2} = \psi$, and $(1+\Phi)\psi = \eta$.

- **Only monolayer (no hBN)**: this is equivalent to setting $a = 0$:

$$1 - (1+\Phi)\psi = \pm(1 + (1+\Phi)\psi).$$

Taking the positive sign solution:

$$1 - (1+\Phi)\psi = 1 + (1+\Phi)\psi,$$

$$(1+\Phi)\psi = 0 \text{ with } \psi = 0 \text{ not possible},$$

$$\Phi = -1,\ i\sigma q = -\omega\varepsilon_0\varepsilon_1,\ q = \frac{i\omega\varepsilon_0\varepsilon_1}{\sigma} = 2q_{\text{monolayer}}.$$

- **Heterostructure:**

$$e^{-2ima}(1-\eta)^2 - e^{2ima}(1+\eta)^2 = 0,$$

$$e^{-ima}(1-\eta) = \pm e^{ima}(1+\eta),$$

$$\eta = \frac{\mp e^{ima} + e^{-ima}}{\pm e^{ima} + e^{-ima}}.$$

We take the positive sign and work out the $a \to 0$ limit:

$$\eta = -i\tan(ma),$$

$$\left(1 + \frac{i\sigma q}{\omega\varepsilon_0\varepsilon_1}\right)\frac{im\varepsilon_1}{k\varepsilon_2} = -i\tan(ma),$$

$$\frac{im\varepsilon_1}{k\varepsilon_2} + \frac{i\sigma m}{\omega\varepsilon_0\varepsilon_2} = -\tan(ma),$$

$$\frac{1}{q} - \frac{1}{q_{\text{monolayer}}} = -\frac{a\varepsilon_2}{\varepsilon_1}\frac{\tan(ma)}{ma} \sim -\frac{a\varepsilon_2}{\varepsilon_1},$$

$$q = \frac{1}{\frac{1}{q_{\text{monolayer}}} - \frac{a\varepsilon_2}{\varepsilon_1}} = \frac{\varepsilon_1}{\frac{\sigma}{i\omega\varepsilon_0} - a\varepsilon_2},$$

$$\sigma = -i\omega\varepsilon_0\varepsilon_{\text{m}}t_{\text{monolayer}},$$

$$q = -\frac{2\varepsilon_1}{\varepsilon_2 h + 2\varepsilon_{\text{m}}t_{\text{monolayer}}}.$$

To calculate the minimum thickness of the spacer, we set the denominator to zero:

$$h_{\text{cutoff}} = -\frac{2\ Re\{\varepsilon_{\text{m}}\}t_{\text{monolayer}}}{\varepsilon_2}.$$

Therefore, when utilizing this analytical theory and applying the small-thickness approximation in the complex-β plane with a real ω, the cutoff length is independent of the permittivity of the substrate and superstrate, and it depends only on the properties of the spacer. We identify the following cutoff thicknesses for different spacer materials: 5.33 nm for air, 2.64 nm for PDMS, and 1 nm for hBN. Above these values, the effective total permittivity becomes positive, and the structure can no longer support the usual TM mode. The TE mode, in contrast, has no cutoff.

Supplementary Section S6. Influence of the insulator spacer on the guided modes for the complex-*ω* and complex-*β* approaches

The dependence of the TE guided mode on the spacer thickness in the complex-β approach agrees with the complex-ω one, as shown in Supplementary Figure S7a. However, the effect of increasing the spacer thickness on the TM mode in the complex-β approach is clearly different from the complex-ω approach, as shown in Supplementary Figure S7b. To clarify this difference, we evaluate the derivative of the propagation constant with respect to the spacer thickness, $d\beta/dh$, which has the same sign as β^2 according to the previous derivation. When considering the complex-ω approach, where β takes a real value, the quantity $d\beta/dh$ is found to be positive. This indicates that the derivative increases as the thickness of the spacer increases, thereby positively impacting the TM mode. Conversely, in the complex-β approach, where β is primarily an imaginary number, the derivative $d\beta/dh$ is negative. This suggests that, as the spacer thickness increases, the derivative decreases, exerting an opposite influence on the TM mode compared to solving the mode in the complex-ω plane.

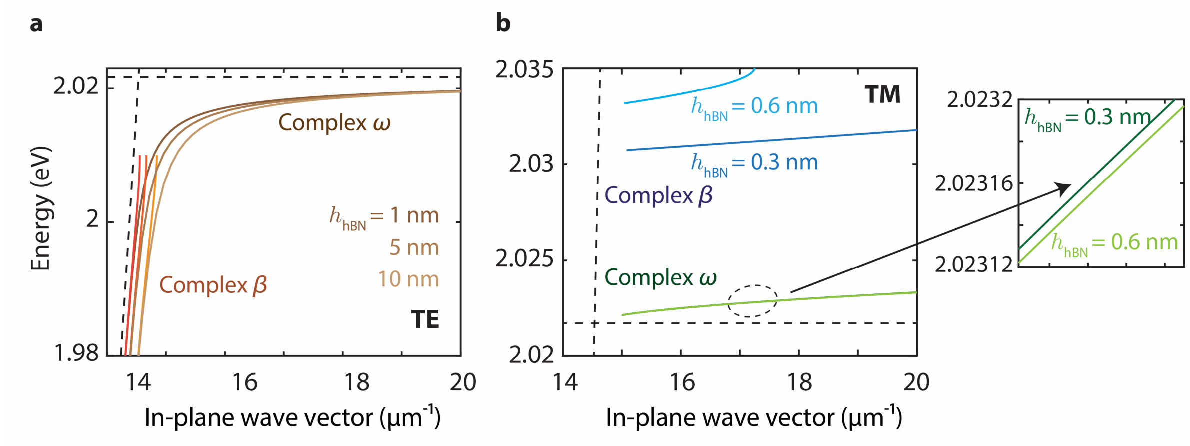

Supplementary Figure S7 | Spacer thickness dependence of the guided modes using the complex-*ω* and complex-*β* approaches. a, Comparison of the TE mode in the complex-ω and complex-β approaches for a heterostructure with varying hBN spacer thicknesses of 1 nm, 5 nm, and 10 nm. **b**, TM-mode dispersion in the complex-ω and complex-β approaches for spacer thicknesses of 0.3 nm and 0.6 nm.
